# Low-Dose Ionizing Radiation Affects Mesenchymal Stem Cells via Extracellular Oxidized Cell-Free DNA: A Possible Mediator of Bystander Effect and Adaptive Response

**DOI:** 10.1155/2017/9515809

**Published:** 2017-08-22

**Authors:** V. A. Sergeeva, E. S. Ershova, N. N. Veiko, E. M. Malinovskaya, A. A. Kalyanov, L. V. Kameneva, S. V. Stukalov, O. A. Dolgikh, M. S. Konkova, A. V. Ermakov, V. P. Veiko, V. L. Izhevskaya, S. I. Kutsev, S. V. Kostyuk

**Affiliations:** ^1^Research Centre for Medical Genetics (RCMG), Moscow 115478, Russia; ^2^V. A. Negovsky Research Institute of General Reanimatology, Moscow 107031, Russia; ^3^Bach Institute of Biochemistry and Russian Academy of Sciences, 33 Leninskii Ave., Moscow 119071, Russia; ^4^N. I. Pirogov Russian National Research Medical University, Moscow 117997, Russia

## Abstract

We have hypothesized that the adaptive response to low doses of ionizing radiation (IR) is mediated by oxidized cell-free DNA (cfDNA) fragments. Here, we summarize our experimental evidence for this model. Studies involving measurements of ROS, expression of the NOX (superoxide radical production), induction of apoptosis and DNA double-strand breaks, antiapoptotic gene expression and cell cycle inhibition confirm this hypothesis. We have demonstrated that treatment of mesenchymal stem cells (MSCs) with low doses of IR (10 cGy) leads to cell death of part of cell population and release of oxidized cfDNA. cfDNA has the ability to penetrate into the cytoplasm of other cells. Oxidized cfDNA, like low doses of IR, induces oxidative stress, ROS production, ROS-induced oxidative modifications of nuclear DNA, DNA breaks, arrest of the cell cycle, activation of DNA reparation and antioxidant response, and inhibition of apoptosis. The MSCs pretreated with low dose of irradiation or oxidized cfDNA were equally effective in induction of adaptive response to challenge further dose of radiation. Our studies suggest that oxidized cfDNA is a signaling molecule in the stress signaling that mediates radiation-induced bystander effects and that it is an important component of the development of radioadaptive responses to low doses of IR.

## 1. Introduction

Human beings are constantly exposed to background sources of IR both of natural (terrestrial and cosmic) and artificial origin (nuclear energy, nuclear accidents, radiation for medical purposes) [1]. The use of IR in research, industry, homeland security, and contemporary medicine is continuously growing and increasing the potential for human exposures [2]. However, the biological effects of low-dose ionizing radiation (LDIR) exposure are still not adequately understood. It is possible that even if there is a potential beneficial hormetic effect, there might still be risks of negative effects that have not been detected [3]. Although there are many published reports available, the understanding of fundamental biological processes and signaling pathways involved in the response to LDIR in human cells is still inconsistent and not fully conclusive [2]. A number of epidemiological studies are available for LDIR exposures below 0.1 Gy on stochastic effects such as cancer incidence and effects on heredity [4, 5], and it was reported that 0.06 Gy of LDIR exposure might increase the risk of brain cancer threefold [6]. It is well accepted that one of the major problems in radiation research is how to extrapolate the data obtained for high-dose IR exposures to the LDIR range (0.1 Gy and less). There is a “linear, no-threshold” hypothesis [7] according to which even the smallest doses of IR could potentially increase the cancer risk. However, the evidence for nonlinearity in biological effects of LDIR is growing [8, 9]. The nontargeted effects of IR, such as radioadaptive responses (RAR), radiation-induced bystander effects (RIBE), and LDIR hypersensitivity, add to the uncertainties of assessing the biological effects of LDIR.

The effects of information transfer from irradiated (target) cells to adjacent, nontargeted cells (RIBE) have been observed for a number of damaging agents of both physical and chemical nature in many types of eukaryotic cells and cover a variety of physiological effects including genomic instability, cell death, and/or RAR [10]. RIBE and RAR are closely interconnected biologically and have many similarities and characteristic features [10–12]. There are three possible pathways of signal transfer from the irradiated cell to the bystander cell: through direct cellular contact with the formation of common membranous structures, through interaction involving gap junctions, or via signals released to the culture medium of the irradiated cells [13], a pathway typical for the RIBE induced by radiation with low linear energy transfer [14]. Many candidate molecules, mainly soluble proteins, have been proposed as mediators of bystander signaling [15, 16].

Research on the role of cell-free DNA (cfDNA) circulating in the blood of healthy persons and patients has led to the hypothesis that oxidized cfDNA (cfDNAox) released from dying cells could mediate RIBE and RAR, and further information on our own research on this subject can be found here [17–20]. We researched the bystander effect in various cell types including G0 lymphocytes of peripheral blood [17] and HUVECs [20]. As we have showed previously, one of the known markers for irradiation-induced chromatin rearrangement, the position of pericentromeric loci of chromosome 1 (1q12) [21], undergoes the same change after 10 cGy of IR and when treated with cfDNAox from the medium from irradiated cells (cfDNAoxR) [18].

Stem cells are undifferentiated cells that have a potential for unlimited division and differentiation into many types of cells. As they have a longer life span, they are more likely to accumulate mutations and lead to cancer [22]. IR can affect the fate of stem cells by inducing DNA damage, arresting the cell cycle or apoptosis, both at genetic and epigenetic levels. Researching the signaling pathways that allow stem cells to survive IR is of importance, and the aim of our work was to assess the development of the RAR to low-dose IR in mesenchymal stem cells (MSCs) and to describe the role of cfDNAox as a stress signaling molecule that mediates RIBE.

## 2. Methods

### 2.1. Cell Culture

MSCs were derived from adipose tissue of patients subjected to surgical operation. To obtain stromal cells, minced adipose tissue was digested with collagenase as described previously [23]. Tissue samples were mechanically disrupted in Dulbecco's Modified Eagle medium (DMEM) (Paneko, Moscow) containing 250 *μ*g/ml gentamycin, 60 U/ml penicillin, and 60 U/ml streptomycin (Paneko). Cells were dissociated by incubation with 0.04% collagenase (Sigma) in DMEM with 10% fetal bovine serum (FBS) (PAA, Austria) at 37°C for 16 h. Cells were centrifuged at 200*g* for 10 min, transferred into slide flasks and cultivated at 37°C in AmnioMax Basal Medium with AmnioMax Supplement C100 (Gibco). Cultures were split no more than four times before experiments.

MSCs were characterized by standard markers using fluorescence-activated cell sorting (FACS): MHC molecules (HLA-ABC+) and adhesion molecules (CD44+, CD54 (low), CD90+, CD106+, CD29+, CD49b (low), and CD105); however, they were negative for hematopoietic markers (CD34−, CD45−, and HLA-DR−) and the marker CD117 (Dominici et al. 2006). Moreover, cells differentiated into adipocytes in the presence of inducers in a kit for adipogenic differentiation (STEMCELL Technologies). Ethical approval for the use of MSCs was obtained from the Regional Committees for Medical and Health Research Ethics (approval number 5).

### 2.2. Irradiation of Cells

MSCs were irradiated in a growth medium at 20°C using a pulsed Röntgen radiation unit (ARINA-3, Spectroflash, Russia). The voltage on the X-tube was ∼160 kV (∼60 keV), peak energy in the spectrum was 60 keV, and dose rate was 10 cGy/min. Nonirradiated cells were used as controls.

### 2.3. Flow Cytometry (FACS)

MSCs were washed in Versene solution (PanEco, Moscow, Russia) and then treated with 0.25% trypsin, washed with medium, and suspended in PBS. Cells were fixed with paraformaldehyde (PFA, Sigma, 2%, 37°C, 10 min), washed three times with 0.5% BSA-PBS, and permeabilized with 0.1% Triton X-100 in PBS (15 min, 20°C) or with 90% methanol (3 h, 4°C). The cells were washed 3× with 0.5% BSA-PBS and labeled with primary antibodies (1 *μ*g/ml) for 2 h (4°C) and then washed 3× with 0.5% BSA-PBS. The following antibodies were used: *γ*H2AX-Dylight488 (pSer139) (NB100-78356G, Novus Biologicals); NOX4 (Sc-30141, Santa Cruz Biotechnology); 8OHDG (Sc-66036, Santa Cruz Biotechnology); BRCA2 (NBP1-88361, Novus Biologicals); PCNA (ab2426, Abcam); Ki-67FITC (sc-23900 FITC, Santa Cruz Biotechnology); and BCL2 (Sc-783, Santa Cruz Biotechnology). Cells were then incubated for 2 h (20°C) with FITC-conjugated goat anti-rabbit IgG (Sc-2012, Santa Cruz Biotechnology) or goat anti-mouse IgG (Sc-2010, Santa Cruz Biotechnology). To quantify the background fluorescence, we stained portions of the cells with secondary FITC-conjugated antibodies only. To quantify DNA, cells were treated with propidium iodide (PI) and RNase A. The cells were analyzed using a CyFlowSpace flow cytometer (Partec, Germany).

### 2.4. Annexin V Binding Assays

Cells were detached, washed with PBS, and treated with annexin V-FITC and PI in buffer (10 mM HEPES, pH 7.4, 140 mM NaCl, 2.5 mM CaCl_2_) at 20°C for 15 min and immediately analyzed using an automated cell counter (Countess II FL, Thermo Fisher) or FACS (CyFlow Space).

### 2.5. ROS Assays

#### 2.5.1. FACS ROS Assay

After irradiation or treatment with cfDNA, cells were washed with PBS and incubated with 10 *μ*M H2DCFH-DA (Invitrogen) at 37°C in the dark for 20 min. Cells were detached, washed with PBS, and immediately analyzed by FACS.

#### 2.5.2. Fluorescence Microscopy

Cells were grown in slide flasks and washed in PBS. Then, 10 *μ*M H2DCFH-DA (Molecular Probes/Invitrogen, CA, USA) was added for 20 min, and cells were washed 3× with PBS and immediately photographed.

#### 2.5.3. Plate ROS Assay

Cells were grown to 80–90% confluency in 96-well plates (Nunclon, Germany). After irradiation or treatment with DNA, cells were incubated with 10 *μ*M H2DCFH-DA (Invitrogen) at 37°C in the dark. Fluorescence was measured with *λ*ex = 503 nm and *λ*em = 524 nm (EnSpire, PerkinElmer, Finland).

### 2.6. Fluorescence Microscopy

Cells were grown in slide flasks, fixed in 2% PFA (4°C, 20 min), washed with PBS, and then permeabilized with 0.1% Triton X-100 in PBS (15 min, 20°C), followed by blocking with 0.5% BSA in PBS (1 h, 4°C), and incubated overnight with rabbit polyclonal antibody against LC3 (Epitomics, Cambridge, MA), *γ*H2AX (pSer139), or NF-*κ*B (p65) (Abcam). After washing with 0.01% Triton X-100 in PBS, cells were incubated for 2 h (20°C) with FITC goat anti-rabbit IgG, washed with PBS, and then stained with DAPI or PI. Nuclear fragmentation was examined in cells washed and stained with Hoechst 33342 (Sigma, 10 *μ*g/ml) for 10 min at 37°C. Images were obtained using an AxioScope A1 microscope (Carl Zeiss).

### 2.7. Quantification of mRNA Levels

Total mRNA was isolated using RNeasy Mini kits (Qiagen, Germany), treated with DNAse I, and reverse transcribed by a Reverse Transcriptase kit (Sileks, Russia). The expression profiles were obtained using qRT-PCR with SYBR Green PCR Master Mix (Applied Biosystems). The mRNA levels were analyzed using the StepOnePlus (Applied Biosystems); the technical error was approximately 2%. The following primers were used (Sintol, Russia):

*NOX4* (TTGGGGCTAGGATTGTGTCTA; GAGTGTTCGGCACATGGGTA);

*BCL2* (TTTGGAAATCCGACCACTAA; AAAGAAATGCAAGTGAATGA);

*BCL2A1* (TACAGGCTGGCTCAGGACTAT; CGCAACATTTTGTAGCACTCTG);

*BCL2L1* (CGACGAGTTTGAACTGCGGTA; GGGATGTCAGGTCACTGAATG);

*CCND1* (TTCGTGGCCTCTAAGATGAAGG; GAGCAGCTCCATTTGCAGC);

*CDKN2A* (ATGGAGCCTTCGGCTGACT; GTAACTATTCGGTGCGTTGGG);

*BRCA1* (GGCTATCCTCTCAGAGTGACATTTTA; GCTTTATCAGGTTATGTTGCATGGT);

*BIRC2* (GAATCTGGTTTCAGCTAGTCTGG; GGTGGGAGATAATGAATGTGCAA);

*BIRC3* (AAGCTACCTCTCAGCCTACTTT; CCACTGTTTTCTGTACCCGGA);

*BAX* (F: CCCGAGAGGTCTTTTTCCGAG, R: CCAGCCCATGATGGTTCTGAT);

*BRCA2 (F:* CCTCTGCCCTTATCATCACTTT, R: CCAGATGATGTCTTCTCCATCC);

*TBP (reference gene)* (F: GCCCGAAACGCCGAATAT, R: CCGTGGTTCGTGGCTCTCT).

The standard curve method was used for the quantification of RNA levels.

### 2.8. Quantitation of 8-OxodG

DNAs were dissolved in 20 *μ*l HPLC-purified water and digested for 1 h at 37°C using 0.5 *μ*l of DNase I (2000 U/*μ*l), 2.3 *μ*l 100 mM MgCl_2_, and 0.5 *μ*l 1 M Tris-HCl (pH 7.4). After adjusting the pH to 5.2 with 0.5 *μ*l of 3 M sodium acetate (pH 5.2), the DNA was further digested with 1 *μ*l of NP1 (1 unit/*μ*l) for 1 h followed by neutralization with 2.3 *μ*l of 1 M Tris-HCl (pH 8.0); 0.5 *μ*l of alkaline phosphatase (1 unit/*μ*l) was added and further incubated for 1 h. Quantitative analysis of 8-oxodG was performed using electrospray ionization mass spectrometry (ESI-MS/MS) on an AB SCIEX 3200 Qtrap machine. The sensitivity of this assay was one molecule of 8-oxodG per 10^7^ molecules of dG.

### 2.9. DNA Oxidation In Vitro

Genomic DNA was isolated from MSCs by phenol-chloroform extraction. Hydrolysis by DNAse I (Invitrogen, USA) was performed until the maximal length of the DNA fragments was below 15 kb. The resulting DNA solution (100 *μ*g/ml) was combined with 300 mM H_2_O_2_ under UV light (312 nm), 30 min, 25°C (gDNAox). The modified DNA was precipitated with 2 volumes of ethanol in the presence of 2 M ammonium acetate, washed twice with 75% ethanol, and then dried and dissolved in water. DNA concentrations were assessed by UV spectrophotometry.

### 2.10. Plasmid Construction

Plasmid pEGFP-C1 that contains the *EGFP* gene (http://www.bdbiosciences.com, GenBank accession number U55763) was used as vector. The DNA fragment to be inserted was synthesized and consisted of 59 base pairs flanked with BamHI restriction sites (italics) and containing the poly-G (underlines) sequence.


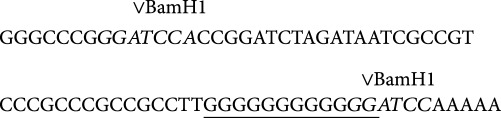


In order to obtain the DNA fragment, PCR with primers GF_601 gggcccgggatccaccggatctagataatcgccgtcccgcccgccgcctt and C10 tttttggatccccccccccccaaggcggcgggcgggacggcga was used.

The fragment was purified by agarose gel electrophoresis and treated with BamHI. The vector pEGFP-C1 was treated with BamHI and added to the DNA fragments with subsequent ligation with T4 DNA ligase. Competent *E. coli* (strain JM110) were then transformed and grown on LB with agarose and kanamycin (50 *μ*g/ml). The clones were analyzed by PCR with oligonucleotides R_SEQ_N and SEQ_C in order to confirm the insertion of the DNA fragment (SEQ_C = catggtcctgctggagttcgtg, R_SEQ_N = caataaacaagttaacaacaacaattgc). Selected clones were grown in liquid medium and plasmids were isolated. After confirmation of the designed DNA sequence by sequencing, the plasmids were extracted using an Invisorb Plasmid Maxi Kit (http://www.invitek.de).

### 2.11. Comet Assays

A cell suspension in low-melting-point agarose was dropped onto slides precoated with 1% normal-melting-point agarose. The slides were placed in a solution (10 mM Tris-HCl, pH 10, 2.5 M NaCl, 100 mM EDTA, 1% Triton X-100, 10% DMSO, 4°C, 1 h) and then in electrophoresis buffer (300 mM NaOH, 1 mM EDTA, pH > 13). Electrophoresis was performed for 20 min at 1 V/cm, 300 mA. The slides were fixed in 70% ethanol and stained with SYBR Green I (Invitrogen, USA).

### 2.12. Statistics

All the reported results were reproduced at least three times as independent biological replicates. In FACS, the median of signal intensities was analyzed. The figures show the mean and standard deviation (SD) values. The significance of the observed differences was analyzed with nonparametric Mann–Whitney *U* tests. *p* values < 0.05 were considered statistically significant and are marked in the figures with ∗. Data were analyzed with StatPlus 2007 professional software (http://www.analystsoft.com/).

## 3. Results

### 3.1. Experimental Design

This study was performed using human MSC lines obtained from different donors and characterized by their CD marker expression (Table 1). We first demonstrated that treatment of MSCs with LDIR (10 cGy) increases the level of 8-oxodG in cfDNA obtained from the culture medium 2–2.5-fold. We also constructed a plasmid containing a DNA fragment that contains a (G)n repeat that is easy to oxidize, which penetrates into the cytoplasm of MSCs when they are irradiated as shown by fluorescence microscopy. In the second series of experiments, we compared the effects of various types of oxidized cfDNA with those of LDIR on the levels of ROS production, DNA breaks, and expression levels of several adaptive response genes. Unless otherwise stated, the IR dose was 10 cGy and the concentration of oxidized cfDNA fragments (cfDNAox and cfDNAoxR) added to cultures was 50 ng/ml with an exposure time of 5 min up to 24 h.

### 3.2. Low-Dose Radiation Causes Oxidation of cfDNA In Vitro

The concentrations of cfDNA in the growth medium used here are 12 ± 2 ng/ml [24]. Previously published studies have reported that genomic DNA (gDNA) from cultured cells contains ∼0.1 to 0.5 8-oxodG per 10^6^ nucleotides [25, 26]. cfDNA in the medium used here contains ∼1 to 5 8-oxodG per 10^6^ nucleotides.

**Figure 1:**
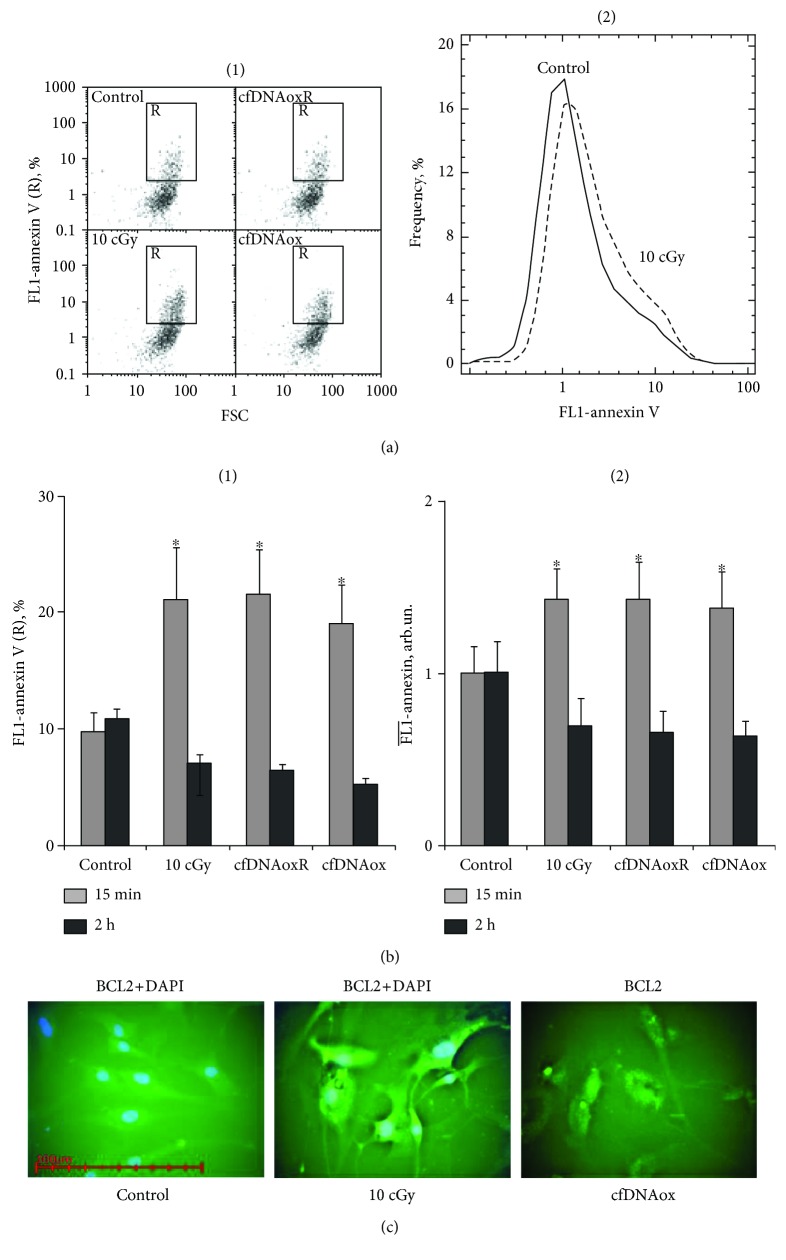
Low-dose radiation causes a strong antiapoptotic response. (a) (1) Flow cytometric enumeration of cells with signs of early apoptosis by FL1 versus FSC. R: gated area, annexin V-positive cells in total population; (2) distribution of fluorescence intensities of cells stained with annexin V-FITC. (b) Signal intensity of FL1-R (1) and average signal intensity of FL1-annexin V-FITC (2) in irradiated (10 cGy, 15 min and 2 h after exposure) and exposed to cfDNAox and cfDNAoxR (50 ng/ml, 15 min and 2 h) cells (flow cytometry). (c) Fluorescence microscopy of irradiated and exposed to cfDNAox MSCs stained with BCL2 (anti-BCL2-antibody and secondary FITC-conjugated antibodies) and DAPI (40×), ^∗^*p* < 0.05.

LDIR can lead to death of a part of the cell population during the first min of exposure. The amount of apoptotic cells after irradiation was assessed using the marker of apoptosis annexin V. 10–20 min after irradiation, cells exhibited signs of apoptosis, and the fraction of apoptotic cells increases 2–2.5-fold (Figure 1(b)). Cell death leads to an increase in the cfDNA concentration in the medium; the concentration 20 min after irradiation with 10 cGy averaged 40 ± 5 ng/ml. IR causes oxidative stress [27] and 10 cGy of LDIR increased the level of 8-oxodG in cfDNA up to 100–200 8-oxodG per 10^6^ nucleotides 20 min after irradiation. The latter will be referred to here as cfDNAoxR.

CG-rich cfDNA fragments are prone to oxidation. We further investigated if the CG-rich oxidized cfDNA penetrates into cells after treating them with 10 cGy of IR.

### 3.3. Oxidized cfDNA Penetrates into Cells Treated with 10 cGy of I∗R

A plasmid containing a marker GFP gene and (G)n repeats that are easy to oxidize was constructed, and its penetration into cells was investigated by fluorescence microscopy and flow cytometry. The cells were treated with plasmids in two ways: (1) the plasmid was added to the medium at a concentration of 100 ng/ml and the cells were then incubated for 24 h and (2) cells were irradiated directly after adding the plasmid to the medium. After cultivation, cells were imaged with the same exposure and magnification. Cells treated by method (1) exhibited weak fluorescence in the cytoplasm, while cells treated by method (2) had a higher level of fluorescence which indicates penetration of the plasmids into the cytoplasm (Figure 1(a)).

As CG-rich oxidized cfDNA penetrates into cells, it might mediate early responses to LDIR (10 cGy).

### 3.4. Oxidized cfDNA Mediates Early Responses to LDIR (10 cGy)

As we showed previously, GC-rich cfDNA can play a role of a signaling molecule in RIBE when lymphocytes from peripheral blood are exposed to LDIR [17]. We hypothesized that oxidized GC-rich cfDNA fragments can be mediators of rapidly repaired DNA breaks in cells exposed to LDIR.

cfDNA from medium of cells 15 min after irradiation was added to MSCs. As the cfDNA in these conditions contains a high level of oxidized bases, model fragments of oxidized DNA were prepared (Table 2) which allowed us to exclude the effect of other factors such as level of methylation and differences in sequence.

cfDNAox fragments were prepared by treatment of gDNA with H_2_O_2_ and Fe^2+^/EDTA and the level of 8-oxodG was assessed (Table 2). Intact genomic DNA contains less 8-oxodG than the threshold of sensitivity of the analysis (0.1 per 10^6^ nucleotides), cfDNAox contains ~400 per 10^6^ nucleotides, and cfDNA from irradiated medium (cfDNAoxR) contains ~200 per 10^6^ nucleotides.

The effect of cfDNAox, cfDNAoxR, or LDIR on the levels of ROS and DNA breaks was investigated. In order to confirm the role of DNA oxidation in these processes, cells were also treated with unoxidized genomic DNA and unoxidized cfDNA from the medium of control cells.

#### 3.4.1. cfDNAox Fragments, like LDIR, Induce a Short-Term Increase in ROS Production

**Figure 2:**
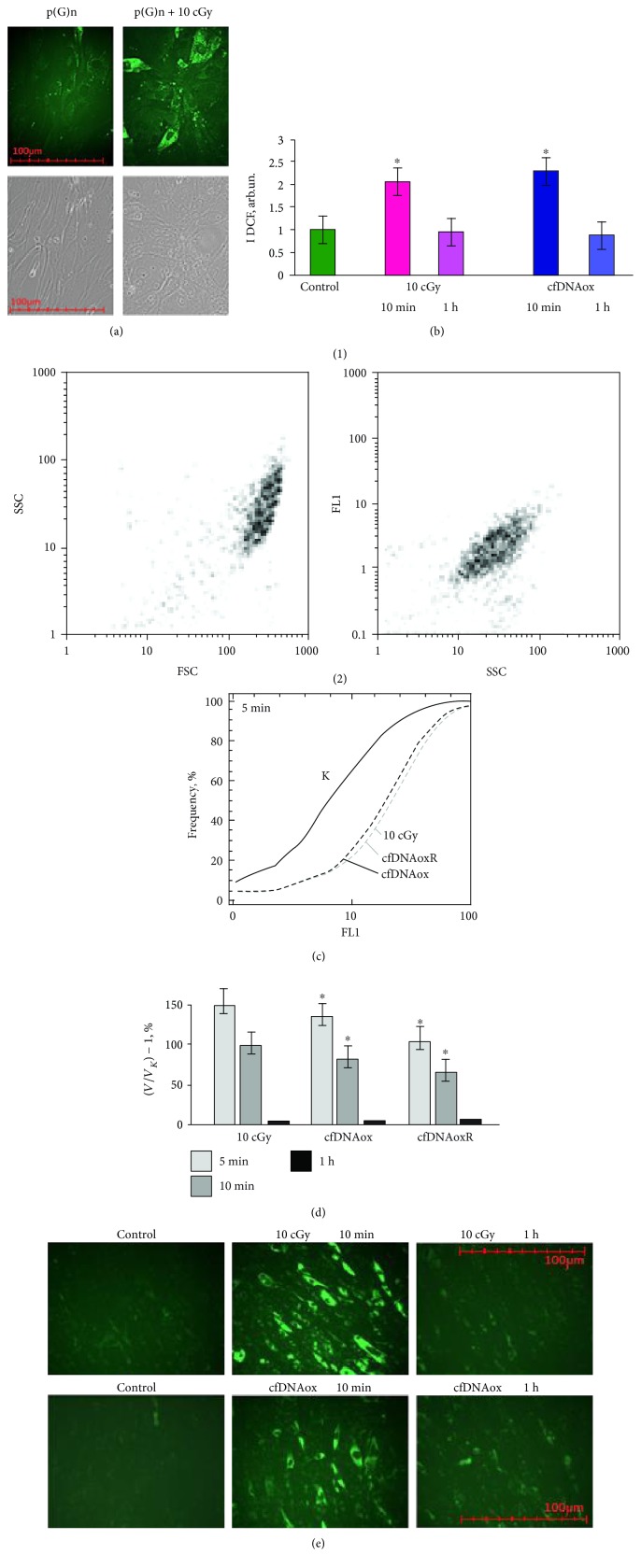
(a) A plasmid containing a marker GFP-gene and containing (G)n repeats that are easy to oxidate penetrates into cells treated with 10 cGy of IR (fluorescent microscopy, 40x). (b–e) Both low-dose radiation (10 cGy) and cfDNAox fragments induce a short-term increase in ROS production. (a, b) Change of DCF fluorescence in the presence of 10 *μ*M of H2DCFH-DA. (c) (1) SSC versus FSC and FL1-DCF versus SSC plots; (2) cumulative histogram of DCF intensity distribution measured by FACS; ^∗^*p* < 0.001, nonparametric *U* test. (d) Ratio of I(DCF) change rate in treated samples (v) to the control samples (vk) (flow cytometry). The light-grey, dark-grey, and black bars correspond to 5, 10, and 60 min time points, respectively. Data points were averaged and represented as mean ± SD for three biological replicates. ^∗^*p* < 0.01, nonparametric Mann–Whitney *U* test. (e) Increase in ROS level measured by fluorescence microscopy 10 min after 10 cGy irradiation or addition of cfDNAox fragments (50 ng/ml); 60 min after exposure ROS returns to control level.

LDIR induces oxidative stress in cells, increasing ROS production. Intracellular ROS level was assessed using H2DCFH-DA (2,7-dichlorofluorescin diacetate) [28, 29] which rapidly penetrates into the cytoplasm where intracellular esterases deacetylate it to form nonfluorescent DCFH [28] which reacts with ROS forming fluorescent DCF [30]. DCF is then detected with a plate reader to provide quantitation of total ROS in the cells. Both IR and oxidized cfDNAox and cfDNAoxR lead to a 2-fold increase of ROS 5–15 min after exposure (Figures 2(b) and 2(d)). However, 30 min after exposure the ROS level decreases and in another 60 min has returned to the control level (Figures 2(b) and 2(d)). These results were confirmed by FACS analysis of the amount of ROS in individual cells (Figures 2(b), 2(c), and 2(d)) and by fluorescence microscopy (Figure 2(e)). Unoxidized gDNA and cfDNA fragments at 50 ng/ml caused an insignificant elevation of ROS synthesis 2 h after addition, most likely due to gradual oxidation of the added fragments. Thus, both intact and oxidized DNA stimulate a short increase in the level of ROS.

Increase in ROS production by cfDNAox fragments can be connected with increased expression of *NOX4*.

#### 3.4.2. cfDNAox Fragments, like LDIR, Induce an Increase in *NOX4* Expression

The main producer of ROS is the NADPH oxidase family (NOX) that includes *NOX* 1–5, DUOX 1, and DUOX2 [31]. *NOX 1–5* are membrane-bound enzyme complexes whose activity is determined by NADPH binding and transfer of an electron to molecular oxygen with formation of a short-living O_2_^−^• that is further transformed into oxygen peroxide (H_2_O_2_) and a hydroxyl radical (•OH) [32]. The *NOX4* expression level is regulated by many factors and changes in response to IR [33]. Since LDIR and cfDNAox had the same effect on ROS production, we expected that they will have a very similar effect on *NOX4* expression.

**Figure 3:**
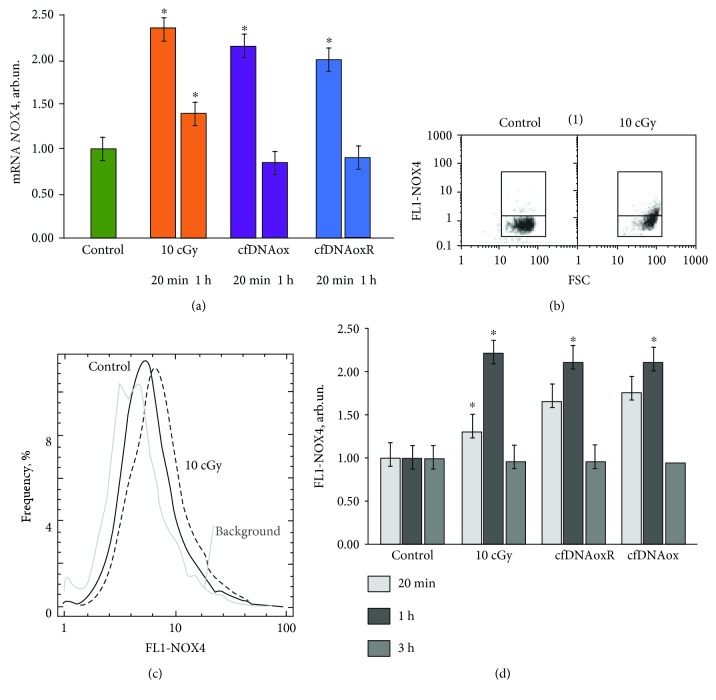
Both low-dose radiation (10 cGy) and cfDNAox and cfDNAoxR (50 ng/ml) fragments induce an increase in *NOX4* expression. (a) Changes in the levels of mRNAs encoding NOX4. NOX4 mRNA in treated cells compared to control (three biological replicates). Reference gene was *TBP*. ^∗^*p* < 0.001, nonparametric *U* test (qRT-PCR). (b) FL1-NOX4 versus SSC plots. Gate R encircles the fraction of MSCs with elevated values of FL1-NOX4 (flow cytometry). (c) Distribution of cells treated with 10 cGy radiation according to the FL1-NOX4 signal strength (flow cytometry). (d) NOX4 level after irradiation with 10 cGy and treatment with 50 ng/ml of cfDNAox and cfDNAoxR and the dynamic change of average fluorescence intensity of NOX4. ^∗^*p* < 0.001, nonparametric *U* test (flow cytometry).

And indeed, LDIR and cfDNAox and cfDNAoxR fragments induce a 2–2.5-fold increase in *NOX4* gene expression after 10–20 min exposure, which after 1 h, it returns to the control level (Figure 3(a)). *NOX4* enzyme expression increases by 30% right after irradiation, and by 60% after adding cfDNAox and cfDNAoxR fragments to the medium, and 1 h after irradiation, the level of *NOX4* expression is 2–2.2-fold increased compared to control, and 3 h after irradiation, it returns to control levels (Figures 3(b) and 3(c)).

The increased level of ROS can induce damage to the cells and cause oxidation of genomic DNA.

#### 3.4.3. cfDNAox and Oxidized cfDNA Fragments Cause Oxidation in Nuclear DNA

**Figure 4:**
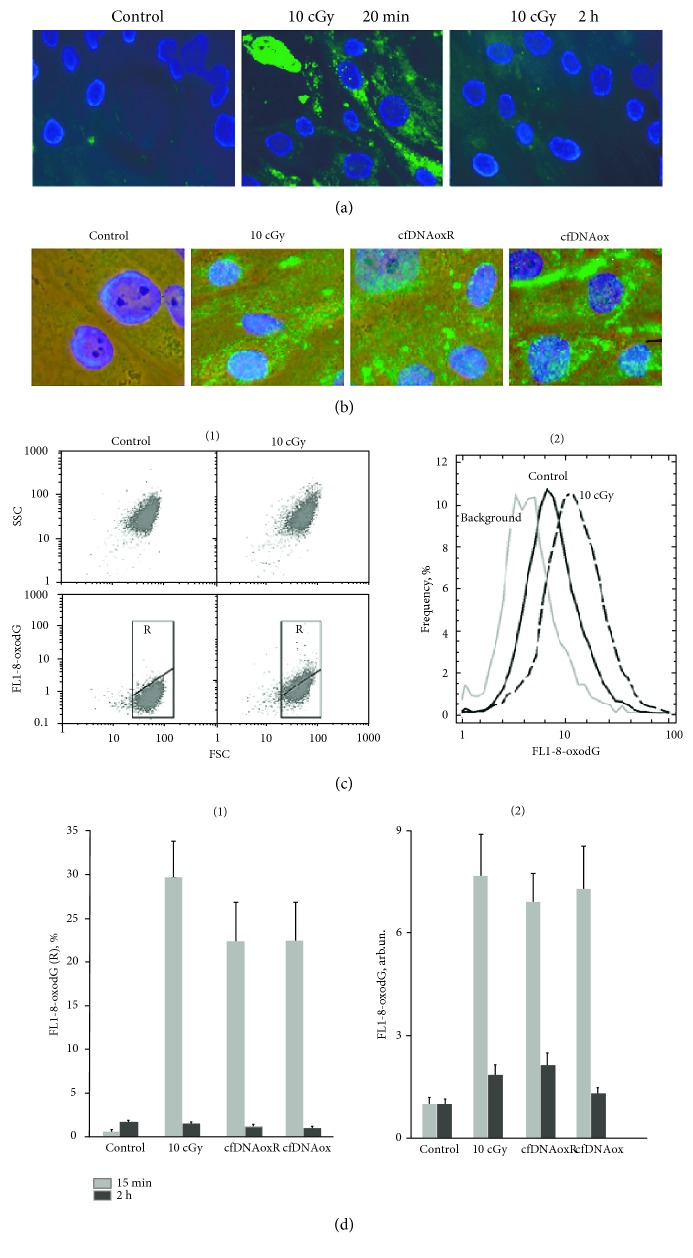
Low-dose radiation and cfDNAox and cfDNAoxR fragments cause oxidation in nuclear DNA. (a, b) Cells stained with antibodies to 8-oxodG (secondary FITC-conjugated antibodies) and DAPI (fluorescence microscopy, 40x (a), 100x (b)). (c, d) Flow cytometry detection of 8-oxodG: (c) (1) analysis of irradiated MSCs stained with antibodies to 8-oxodG FL1-8-oxodG versus SSC plots. Gate R encircles the fraction of MSCs with elevated values of 8-oxodG (secondary FITC-conjugated antibodies); (c) (2) distribution of the cells with varying 8-oxodG contents. (d) (1) Signal intensity of FL1-R; (d) (2) median signal intensity of FL1 (mean value for three independent experiments). ^∗^*p* < 0.01 against control group of cells, nonparametric *U* test.

LDIR, as well as cfDNAox and cfDNAoxR, causes an increase in ROS production that leads to oxidation of nuclear DNA. An FITC-labeled antibody was used to detect 8-oxodG. Control cells did not contain FITC-labeled antibody; there were single cells in the population that had labeled cytoplasm, possibly due to oxidized mitochondrial DNA. Three main types of cells are present after irradiation: (1) with labeled nuclei, (2) with labeled cytoplasm, and (3) both nucleus and the cytoplasm are labeled. Fifteen–20 min after irradiation with 10 cGy, the fluorescent intensity of the cytoplasm and the amount of stained nuclei increased (Figures 4(a) and 4(b)). Two h after irradiation, the staining returns to the control level. CfDNAox and cfDNAoxR fragments have a similar effect; 20 min after treatment, the intensity increases compared to control (Figures 4(a) and 4(b)).

Two subpopulations of cells were detected by flow cytometry; 3–5% of the total population showed high levels of 8-oxodG (Figure 4(c), subpopulation R) and others with low levels of 8-oxodG (Figure 4(c)), cells outside of R). In control cells, the R fraction comprises 3 ± 2% of the total population. The R fraction and its level of intensity increase 8–10-fold 20–30 min after irradiation or treatment with cfDNAox and cfDNAoxR fragments (Figure 4(d) (1)) and after 2 h the intensity returns to control (Figure 4(d) (1)).

The total amount of 8-oxodG fluorescence 20–30 min after irradiation or treatment with cfDNAox and cfDNAoxR fragments increases 7-8-fold (Figure 4(d) (2)) and after 2 h decreases but remains 1.5–2-fold higher than the control (Figure 4(d) (2)). Unoxidized gDNA and cfDNA fragments at 50 ng/ml did not induce oxidative modification of nuclear DNA during 2 h of incubation. Thus, LDIR stimulates ROS production by activation of NOX4 and leads to oxidative modification of nuclear DNA.

Oxidative modification of nuclear DNA can lead to DNA breaks in cell nuclei.

#### 3.4.4. Oxidized cfDNA Fragments, like LDIR, Cause DNA Damage and Breaks in Nuclear DNA

Since oxidation of DNA can cause single- and double-strand breaks [31], we assessed DNA damage by comet assays and flow cytometry for *γ*H2AX labeling. The comet assay allows detection of both single- and double-strand breaks and is widely used to determine the extent of DNA damage [34].

**Figure 5:**
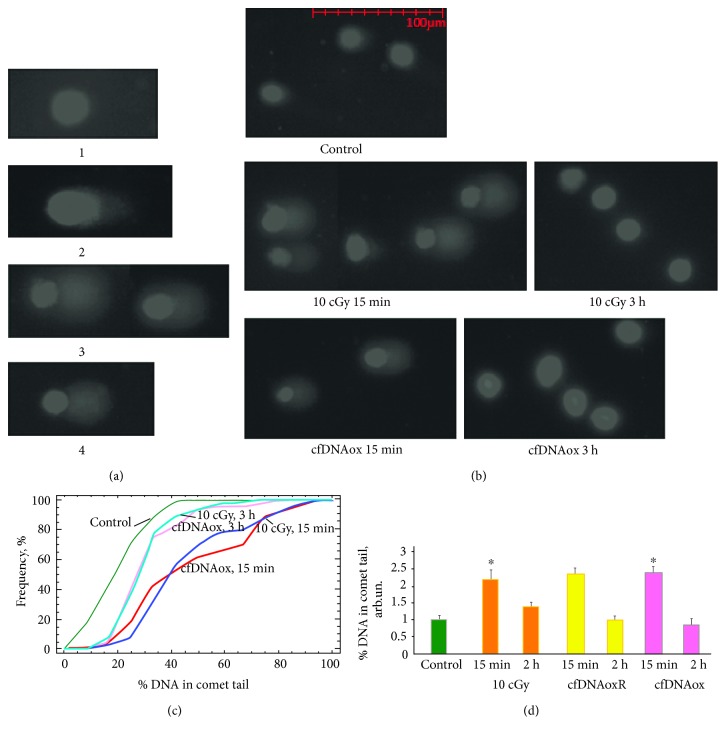
Low-dose radiation and cfDNAox and cfDNAoxR fragments (50 ng/ml) cause DNA breaks in nuclear DNA of exposed MSCs. (a) Different types of nuclei with varying degree of DNA damage (100×). (b) Nuclei of irradiated (10 cGy) and exposed to cfDNAox (50 ng/ml) MSCs, 5 min and 3 h after exposure; (c) cumulative histograms for tail moment of irradiated (10 cGy, 5 min and 3 h) and exposed to cfDNAox (50 ng/ml, 5 min and 3 h) MSCs; (d) percentage of DNA within tails. The significance of differences with the control in the obtained distributions was analyzed by means of the Kolmogorov-Smirnov statistics. ∗ means that *p* < 0.05.

Comet assays were performed 5 min and 3 h after exposure to LDIR (10 cGy) or to cfDNAox and cfDNAoxR fragments. Four types of cells were present in control populations: (1) cells without DNA breaks, (2) cells with few DNA breaks, (3) cells with fragmented DNA, and (4) apoptotic cells with very damaged DNA (Figure 5(a)). Exposure to 10 cGy of radiation, as well as to cfDNAox and cfDNAoxR, stimulated DNA breaks; after 5–20 min of exposure, most cells were of 3 types (Figures 5(b), 5(c), and 5(d)). After 3 h, the amount of cells with damaged DNA was significantly reduced in both cases (Figures 5(b), 5(c), and 5(d)).

**Figure 6:**
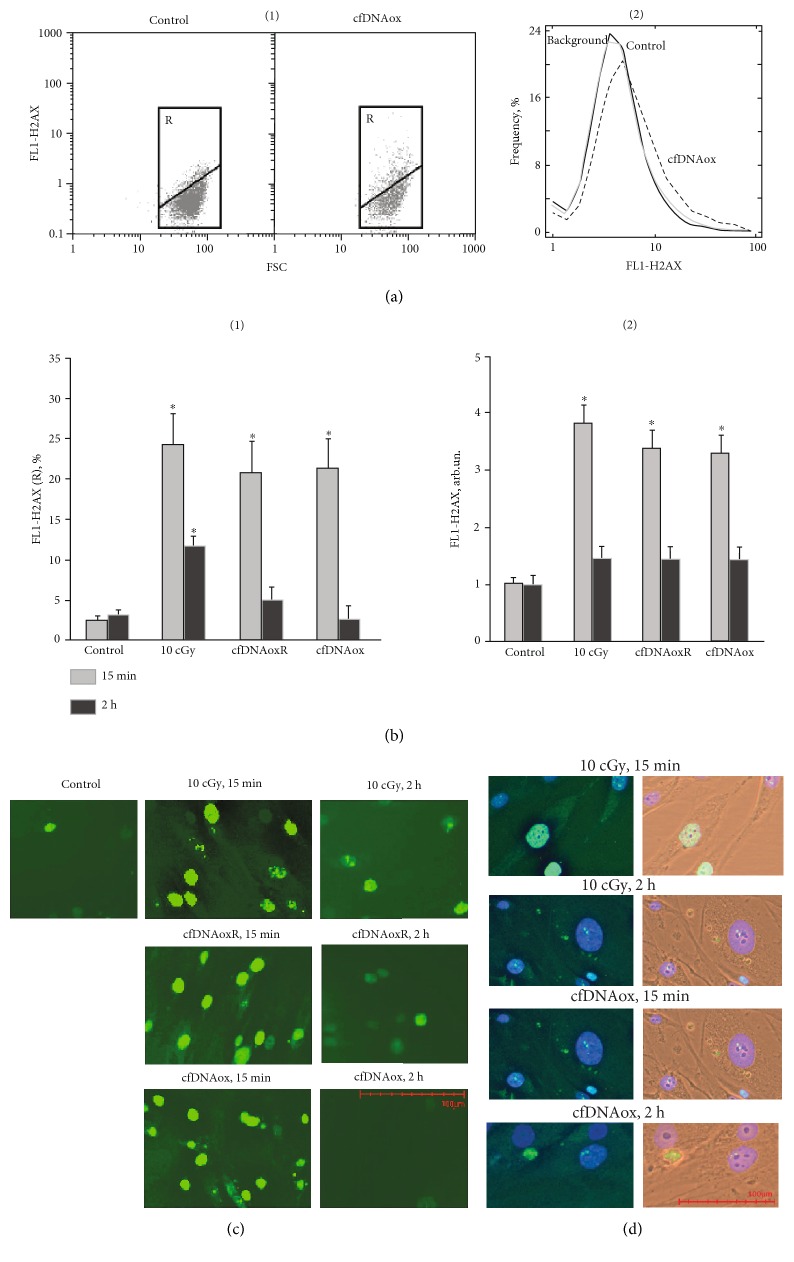
Low-dose radiation and cfDNAox and cfDNAoxR fragments cause DNA breaks and *γ*H2AX foci in nuclear DNA. (a) (1) Flow cytometry detection of DSB in cells exposed to cfDNAox and cfDNAoxR fragments. Cells were processed for immunofluorescence staining with anti *γ*H2AX antibody-DyLight488 (FL1). Gate R encircles the fraction of cells with elevated values of FL1-*γ*H2AX. (2) Distribution of *γ*H2AX fluorescence intensities with varying DSB levels. (b) Median signal intensity of FL1 (mean value for three independent experiments). (c, d) DSB in irradiated cells (10 cGy, 15 min and 2 h after exposure) and cells exposed to cfDNAox and cfDNAoxR (50 ng/ml, 15 min and 2 h, fluorescence microscopy). Cells stained with anti *γ*H2AX antibodies (c) and DAPI (d), ^∗^*p* < 0.05.

DSBs were revealed by immunostaining with antibodies against the histone *γ*H2AX phosphorylated on Ser-139 that rapidly accumulates at DNA loci flanking DSB sites [35]. Cells were fixed and stained with FITC-labeled antibodies for *γ*H2AX and FACS was used for quantitative assessment of the phospho-*γ*H2AX level (Figure 6(a)). Two subpopulations of cells were present, one with a high level of fluorescence (R) and the main fraction with low fluorescence (Figure 6(a)). Control cells from the R subpopulation comprise 3 ± 2% of the total cell count. The amount of cells in R and their intensity of fluorescence increases 7-8-fold 15–30 min after LDIR or treatment with cfDNAox and cfDNAoxR fragments (Figure 6(b)). Two h after 10 cGy of radiation, the level of gamma-H2AX fluorescence of R decreased 2-fold, and after treatment with cfDNAox and cfDNAoxR fragments, it decreased to control levels (Figure 6(b) (1)). The total amount of *γ*H2AX after LDIR or treatment with cfDNAox and cfDNAoxR fragments increases 3.4–3.8-fold, and after 2 h, it returns to control (Figure 6(b) (2)).

FACS allows assessing the average amount of H2AX histone in cells, but these numbers do not always reflect the real degree of DNA damage [36]. To find the reason for the quantitative changes in the level of *γ*H2AX in cells after LDIR or after treating them with cfDNAox and cfDNAoxR, we analyzed fixed cells stained with antibodies for *γ*H2AX by fluorescence microscopy (Figures 6(c) and 6(d)). Exposure to LDIR or addition of cfDNAox and cfDNAoxR fragments to the medium elevates the level of cells with multiple DNA breaks 3–5-fold within 20 min (Figures 6(c) and 6(d)), but after 2 h, this level decreases and only cells containing very few breaks are present (Figure 6(d)). Unoxidized gDNA and cfDNA fragments do not induce DNA breaks during the first 2 h of incubation.

Thus, the damage to the cell nuclei that is induced by LDIR can be mediated by oxidized DNA fragments. cfDNAox penetrates into cells increasing ROS production and leading to oxidative stress and multiple DNA breaks the amount of which decreases 2 h after irradiation or start of incubation with oxidized cfDNA. We researched if the DNA breaks are repaired or that cells with multiple DNA breaks undergo cell death within these 2 h.

#### 3.4.5. Oxidized cfDNA Fragments, like LDIR, Activate Repair of Nuclear DNA

DNA damage induced by IR activates signaling cascades that control DNA repair [37]. Double-strand breaks (DSB) are one of the most dangerous forms of DNA damage. BRCA1 is a nuclear protein that takes part in the regulation of the cell cycle and DSB repair by homologous recombination [38].

**Figure 7:**
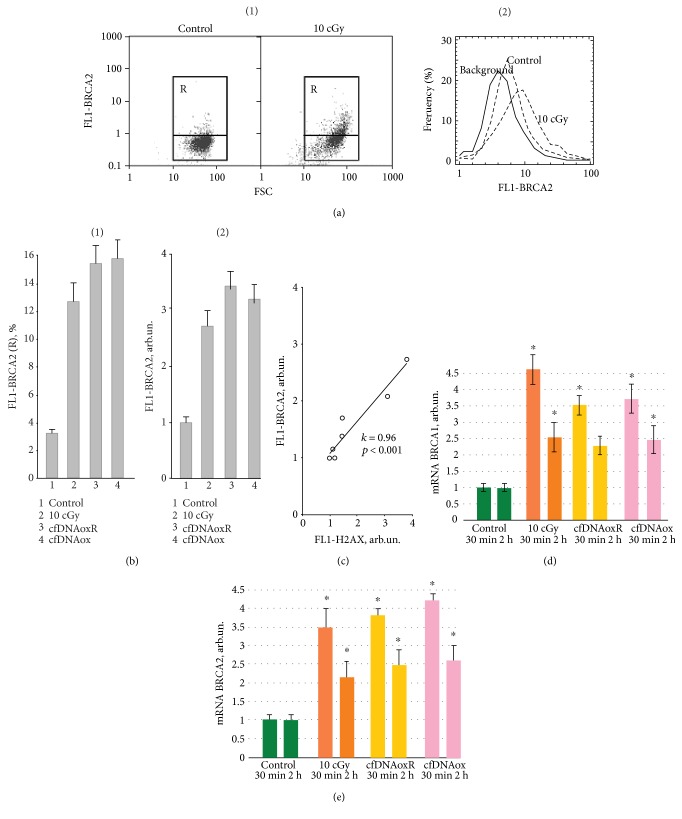
DNA damage induced by low-dose radiation and cfDNAox and cfDNAoxR fragments activates reparation of nuclear DNA. (a) (1) Flow cytometry detection of BRCA2 in irradiated (10 cGy) and control cells stained with anti-BRCA2 antibodies and secondary FITC-conjugated antibodies. Gate R encircles the fraction of MSCs with elevated values of FL1- BRCA2; (2) distribution of cells with varying BRCA2 contents. (b) The signal intensity of FL1-R (1) and average signal intensity of FL1-BRCA2 (2) in irradiated cells (10 cGy, 15 min and 2 h after exposure) and cells exposed to cfDNAox and cfDNAoxR (50 ng/ml, 15 min and 2 h) (flow cytometry). (c) Linear correlation between the levels of *γ*H2AX and BRCA2 (*k* = 0.96; *p* < 0.0001). (d, e) Dependence of the changes in the levels of mRNA BRCA1 (d) and BRCA2 (e) in irradiated cells and cells exposed to cfDNAox and cfDNAoxR (RT-PCR); mRNA level—average expression of genes in treated cells compared to control (for three biological replicates). Reference gene, *TBP*. ^∗^*p* < 0.001, nonparametric *U* test (qRT-PCR).

LDIR or treatment with cfDNAox and cfDNAoxR fragments leads to a 3.5–4.5-fold increment in the level of mRNA transcripts from the *BRCA1* and *BRCA2* genes after 30 min (Figures 7(d) and 7(e)), and after 2 h, this level remains 2–2.5-fold higher than the control level (Figures 7(d) and 7(e)). These results were confirmed at the protein level.  Figure 7(b) is a histogram of BRCA2 protein expression where R is an area of cells with high fluorescence (Figures 7(a) and 7(b)). Thirty min after irradiation or addition of cfDNAox and cfDNAoxR fragments to the medium, the level of BRCA2 in R is increased 4-5-fold (Figure 7(b)). The total BRCA2 level increased 30 min after irradiation with 10 cGy and 2.5–3.5-fold after the addition of cfDNAox and cfDNAoxR fragments (Figure 7(b)) and remained increased 2–2.5-fold 2 h after. Thus, there is a correlation between DSB formation and the level of BRCA2 that reflects the efficacy of DNA repair after irradiation (Figure 7(c)). LDIR or oxidized DNA fragments increase the amount of DSBs but, at the same time, activate DNA repair which leads to the minimization of the amount of breaks in the cell DNA. Unoxidized gDNA and cfDNA fragments at 50 ng/ml did not affect the level of BRCA2 2 h after the start of incubation.

Thus, DNA breaks activate DNA reparation system in the treated cells. As DNA reparation requires time, we were expecting to see a short-term arrest of the cell cycle after treating cells with LDIR or cfDNAox or cfDNAoxR fragments.

#### 3.4.6. cfDNAox and cfDNAoxR Fragments, like LDIR, Cause a Short-Term Arrest of the Cell Cycle and Decrease Proliferation

**Figure 8:**
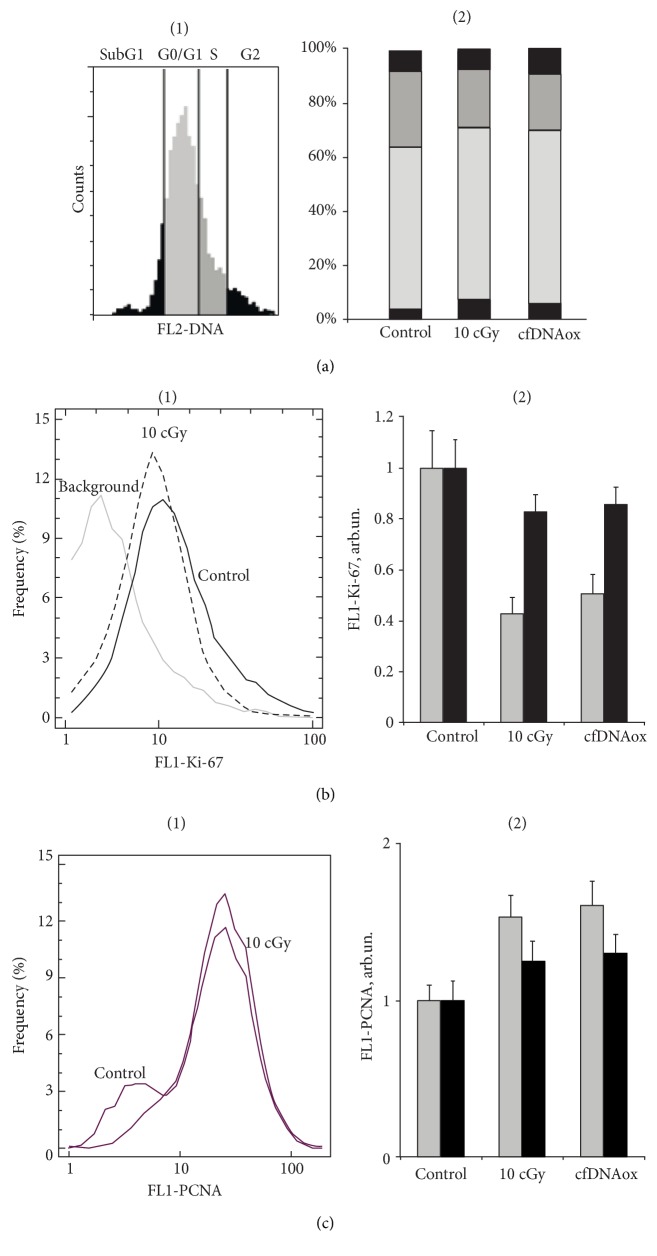
Low-dose radiation and cfDNAox fragments cause a short-term arrest of cell cycle and decreases proliferation. (a) (1) Proportions of cells that contain the amount of DNA characteristic for G1, S, and G2/M phases of the cell-cycle (flow cytometry). (a) (2) Proportions for control cells,10 cGy irradiated cells, and cells treated with oxidized DNA. (b) Dynamics of the change of fraction of proliferating cells (Ki-67+ fraction) in the population (flow cytometry): (1) distribution of cells with varying Ki-67 content; (2) average signal intensity of FL1-Ki-67+ in irradiated cells (10 cGy, 15 min and 2 h after exposure) and cells exposed to cfDNAox (50 ng/ml, 30 min and 2 h). ^∗^Significantly differs from control (*p* < 0.05). (c) Change of PCNA level in control and exposed cells (flow cytometry): (1) distribution of cells with varying Ki-67 contents; (2) average PCNA fluorescence. ^∗^Significantly different from control (*p* < 0.05).

Oxidative stress and DNA damage lead to the arrest of the cell cycle at all stages and block proliferation [39]. We analyzed the effect of low-dose radiation and cfDNAox and cfDNAoxR fragments on the level of proliferation using antibodies to the proliferation markers Ki-67 and PCNA [40] and flow cytometry. All types of cells express Ki-67 and PCNA during all stages of the cell cycle [40]. Cells were stained with propidium iodide in order to assess the total amount of DNA. LDIR or cfDNAox fragments decrease the level of Ki-67 protein; 30 min after treatment, Ki-67 is 40–50% lower than that in the control, and 2 h after, it starts increasing and almost reaches control level (Figure 8(b)).

PCNA is a transcription factor for polymerase Δ that is a part of the DNA repair system. We observed a 40–60% increase of the level of PCNA 30 min after irradiation or treatment of cells with oxidized cfDNA (Figure 8(c)), indicating that repair processes are active and proliferation is decreased. As a decrease of proliferation usually occurs when the cell cycle is arrested [40], we assessed the stage at which the cycle stops after irradiation and treatment with oxidized DNA.

Ten cGy of radiation or addition of cfDNAox and cfDNAoxR fragments to the medium increases the number of cells in G1 and G0/G1 30 min after exposure, and thus, these factors arrest the cell cycle in G1 (Figure 8(a) (2)). Three h after irradiation or start of incubation with oxidized DNA, the cell cycle returns to normal.

Exposure to LDIR or addition of cfDNAox and cfDNAoxR fragments causes a 1.5–2-fold decrease in the level of expression of CCND1 and a 1.5–2-fold increase in expression of *CDKN2* and *CDKN1A* 30 min after the exposure, indicating that the cell-cycle undergoes a short-term arrest. However, 3 h after the exposure, the level of expression of *CCND1* increases 20–25% compared to control and *CDKN2* and *CDKN1A* decrease to control level. Thus, the cell cycle is shortly arrested at the G1-phase and the cells have time for repair of the damage and then they can progress further through the cycle.

Thus, DNA damage leads to a short-term arrest of the cell cycle and activation of DNA reparation. As a result, the level of proliferation, as well as the amount of cells in the population, increases in 2-3 h after treatment. The increased amounts of cells in the population can be a result of low level of apoptosis.

#### 3.4.7. LDIR Causes a Strong Antiapoptotic Response

The amount of apoptotic cells after irradiation or treatment with cfDNAox and cfDNAoxR fragments was assessed using a marker of apoptosis, annexin V, and FACS (Figure 1). After 15 min, cells exhibit signs of apoptosis, and the fraction of apoptotic cells increases ≈2-fold and the apoptosis level 30–40% (Figure 1(b)). Despite that, 2 h after irradiation, the level of apoptosis decreases compared to control (Figure 1(b)). These results were confirmed using an automated cell counter after propidium iodide and annexin V FITC staining; the amount of apoptotic cells increases 3-fold 10 min after irradiation, but decreases 3-fold compared to control 3 h after irradiation.

Bcl2 is a family of proteins that are crucial for cell survival and apoptosis regulation. It includes three groups of interacting and functionally different proteins [41]. Bcl-2 and its 4 relative proteins (BCL-XL, Bcl-W, A1, and Mcl-1) are antiapoptotic, whereas Bax, Bak, Box and Bik, Bad, Bim, Puma, Bid, Noxa, Hrk, Bmf are proapoptotic. Other important factors that decide the cell fate are apoptotic protein inhibitors like BIRC2 and BIRC3 that inhibit the activity of caspase-3, caspase-7, and caspase-9 [42].

Antiapoptotic processes are activated 20 min after irradiation or the addition of cfDNAox and cfDNAoxR fragments to the medium, and the levels of antiapoptotic genes *BCL2*, *BCL2A1 (Bfl-1/A1)*, *BCL2L1 (BCL-X)*, *BIRC2 (c-IAP1)*, and *BIRC3 (c-IAP2)* increase 1.5–2.5-fold (Table 3). The level of proapoptotic *BAX* is increased 20 min after irradiation or treatment with cfDNAox and cfDNAoxR fragments, in agreement with the increased apoptosis level 15 min after exposure. *Bcl2* family genes have increased expression 2–72 h after irradiation or treatment with cfDNAox and cfDNAoxR fragments. Unoxidized gDNA and cfDNA fragments at 50 ng/ml increased the expression level of the antiapoptotic genes *BCL2*, *BCL2A1 (Bfl-1/A1)*, *BCL2L1 (BCL-X)*, *BIRC2 (c-IAP1)*, and *BIRC3 (c-IAP2)* only 20–30% 3 h after the start of incubation. As these fragments caused an extended ROS production and the activation of antiapoptotic genes after only 3 h, the effect is most likely connected to these fragments being gradually oxidized. Flow cytometry and fluorescence microscopy show that Bcl2 protein expression is increased 30 min after irradiation or addition of cfDNAox and cfDNAoxR fragments, and it remains increased 2–72 h after exposure (Figure 1(b)). Thus, the antiapoptotic response to LDIR or addition of oxidized DNA fragments is strong.

#### 3.4.8. cfDNAox Fragments, like LDIR, Activate the Antioxidant Response

**Figure 9:**
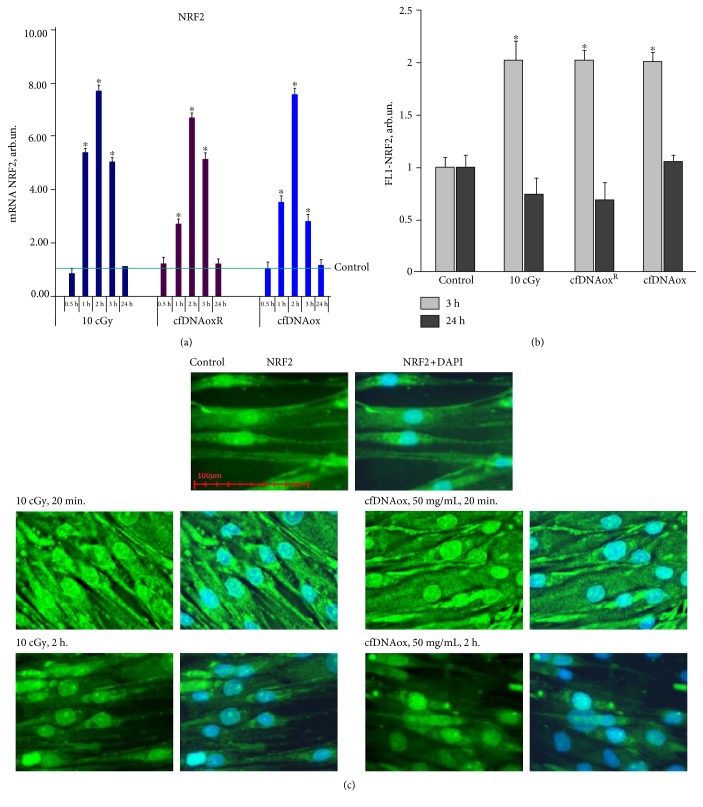
Low-dose radiation and cfDNAox and cfDNAoxR fragments activate antioxidant response. (a) Dependence of changes in the levels of NRF2 mRNA in irradiated cells and cells exposed to cfDNAox and cfDNAoxR on the time after exposure (RT-PCR); mRNA levels—average expression of genes in treated cells compared to controls (three biological replicates). Reference gene, *TBP*. ^∗^*p* < 0.001, nonparametric *U* test (qRT-PCR). (b) Average median signal intensities in cells stained with anti-NRF2-FITC antibodies after various exposures (3 h and 24 h after exposure, flow cytometry). (c) Fluorescence microscopy of irradiated cells and cells exposed to cfDNAox stained with anti-NRF2-FITC antibodies and DAPI.

Oxidative stress caused by an increase of ROS production can activate an antioxidant response in which one of the main regulators is transcription factor NRF2 [43]. One h after LDIR, the level of *NRF2* gene expression increases 5.5-fold. CfDNAox and cfDNAoxR fragments increase the expression of the *NRF2* gene 3-4-fold 1 h after the addition to the medium (Figure 9(a)). Two h after irradiation or cfDNAox and cfDNAoxR fragments, *NRF2* expression is 7-8-fold increased compared to control, and after 3 h, it goes down but still remains 3–5-fold higher than in control cells (Figure 9(a)). The level of protein NRF2 is increased 2-fold after irradiation or addition of cfDNAox and cfDNAoxR fragments (Figure 9(b)), but 24 h after the initial exposure, it returns to control levels (Figure 9(b)).

The key event of antioxidant response development is the translocation of NRF2 to the nucleus. To analyze the effect of oxidized DNA fragments on the location of NRF2, antibodies against NRF2 and fluorescence microscopy were used (Figure 9(c)). When cells are irradiated or treated with cfDNAox and cfDNAoxR fragments, NRF2 expression increases both in the cytoplasm and in the nucleus 1 h after the exposure (Figure 9(c)). Three h after irradiation, NRF2 expression decreases yet the factor remains in the nucleus and can activate antioxidant genes (Figure 9(c)). Unoxidized gDNA and cfDNA fragments (50 ng/ml) did not affect NRF2 expression within 2 h after the start of incubation. Thus, both LDIR and cfDNAox induce a strong antioxidant response.

### 3.5. Oxidized cfDNA Fragments, as well as LDIR, Cause an Adaptive Response

**Figure 10:**
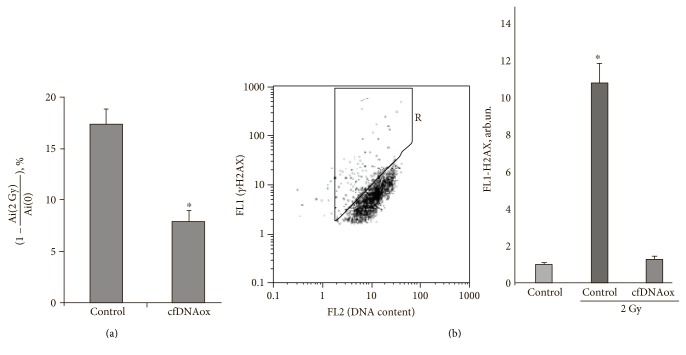
The effect of cfDNAox on the survival of cells and formation of *γ*H2AX foci after exposure to radiation (2 Gy). haMSCs were grown for 3 h in presence of cfDNAox and then irradiated with 2 Gy and grown in fresh media for 48 h more before GG assay. Ai (0), Ai (2 Gy)—absorbance of MTT derivative as normalized to the number of assayed cells in control and irradiated cell populations after preconditioning. ^∗^The differences are significant at *p* < 0.01. (b) (1) Fluorescence-activated cell sorting (FACS) analysis was used to assess the accumulation of gH2AX foci in haMSCs. The plot of FL1 (gH2AX) versus FL2 (DNA content, PI) is shown as drawn for the control cells. The Gate R denotes the fraction of haMSCs, with type 2 nuclei and large number of gH2AX foci. (b) (2) Mean relative fluorescence of the main population of haMSCs (b) after subtraction of the background. Means for the three experiments and SD are shown. ^∗^The difference from the control is statistically significant (*p* < 0.05).

We assessed the effect of oxidized cfDNA and LDIR on the survival of human adipose-derived mesenchymal stem cells (haMSCs) that were subsequently exposed to irradiation at 2 Gy. haMSCs were grown for 3 h in presence of cfDNAox and cfDNAoxR and then irradiated with 2 Gy. In a different experiment, we first irradiated cells with 10 cGy, cultivated them for 3 h, and then irradiated again with a dose of 2 Gy and grew them in fresh media for 48 h more. MTT tests demonstrated a statistically significant decrease in the cell death induced by 2 Gy of irradiation in cells that were pretreated with oxidized cfDNA or preirradiated with 10 cGy (*p* < 0.01; Mann–Whitney test, Figure 10(a)). Moreover, both preconditioning with cfDNAox at 50 ng/ml and preirradiation with 10 cGy 3 h before irradiation with a dose of 2 Gy decrease the proportion of cells containing gamma-H2AX foci (Figure 10(b)).

## 4. Discussion

The predominant hypothesis concerning the origin of cfDNA is that its main source is dead cells [44], but another hypothesis suggests that cfDNA could be actively excreted by living cells [45]. The main reasons for enrichment of the cfDNA pool with oxidized DNA fragments are the death of cells with a high level of DNA oxidation and the GC enrichment of cfDNA compared to total nuclear DNA [13]. The proportion of mitochondrial DNA in cfDNA increases under conditions of oxidative stress [46–48], and this process is relevant as mitochondrial DNA, on average, contains larger amounts of 8-oxodG compared to genomic DNA and thus contributes to the pool of oxidized cfDNA [49].

Increased levels of 8-oxodG in cfDNA can be a sign of oxidative stress, in our case, as a consequence of LDIR. It should be noted that GC-rich fragments within genomic DNA tend to accumulate oxidative damage as well. We have previously demonstrated that chronic exposure to gamma-neutron or tritium *β*-radiation evokes an increase in the content of GC-rich sequences (69% GC) in the transcribed region of human ribosomal repeat (rDNA) in cfDNA from 166 individuals [50]. The reason for this phenomenon is the increased stability of GC repeats to hydrolysis [51]. The transcribed area of rDNA is one of the examples of preferentially oxidized DNA [52]. Thus, in addition to enrichment of cfDNA pools with oxidized DNA of dying cells, the content of 8-oxodG in cfDNA may depend on the somewhat slowed-down degradation in human serum of GC-rich fragments as compared to AT-rich fragments [53].

The cellular response to irradiation depends on a wide variety of factors, but the most important of these is a substantial increase in the level of ROS within a time frame of several seconds to 2–5 min [13]. ROS induces damage to cellular DNA, including the rupture of deoxyribose rings, the appearance of apurinic and apyrimidinic sites, single- and double-strand breaks, DNA protein crosslinks, and formation of oxidized bases [13].

Ionizing low-LET irradiation increases the rate of apoptosis in various cell types within min after irradiation. Dying cells release fragments of chromatin, contributing to the pool of cfDNA and increasing its concentration in the medium. cfDNA from irradiated cells contains significantly larger amounts of the oxidation marker 8-oxodG than cfDNA of control (nonirradiated) cells or cellular DNA of irradiated cells [54]. Here, cfDNA collected from the medium of LDIR (10 cGy)-irradiated cells and DNA oxidized in vitro affects control nonirradiated cells in the same way, indicating that cfDNA released from dying irradiated cells can serve as a stress signal and be a factor in stress signaling of radiation-induced bystander effects and of radioadaptive responses. It is of importance that the cfDNA of nonirradiated cells and unoxidized genomic DNA are not stress signals, as neither of them induces ROS synthesis in control cells.

A variety of studies concern RIBE and RAR based on various parameters of target and bystander cells [10, 54], and we have previously studied the bystander effect in diverse cell types [12, 20, 54]. One marker for irradiation-induced chromatin rearrangement is the position of pericentromeric loci of chromosome 1 (1q12) [20]. Exposure to cfDNA extracted from the culture media of irradiated MSCs leads to similar structural rearrangements of chromatin [55]. All these effects were primarily dependent on an increase in the production of ROS in cells because when a scavenger of ROS, *α*-tocopherol, was added to the medium, they were blocked [56]. We have shown that the addition of cfDNA from the medium of irradiated cells to the medium of endotheliocytes leads to a decrease in the number of cells with DNA breaks [57], an effect similar to that observed when cells were irradiated in low doses. The incubation medium of irradiated cells induces the initial stages of the apoptotic cascade in bystander cells that is accompanied by an increase in the content of ROS within a 6 h time frame [58]. These examples indicate that the patterns of the effects caused by LDIR can be transmitted via the culture medium (or via the extracellular space in the organism) and cfDNA is the most likely molecule to be the stress signal in RIBE. Further evidence for this hypothesis is the fact that cfDNA from the medium of control nonirradiated cells does not produce any of the effects described above, and no adaptive response is observed. Moreover, if the cfDNA from irradiated cells is treated with DNAse I, it loses its ability to cause an adaptive response [27, 28].

The goal of this work was to compare the action of model oxidized DNA fragments (cfDNAox) and cfDNA from the medium of irradiated cells (cfDNAoxR) with those of LDIR (10 cGy) on human adipose-derived MSCs, and we obtained hard evidence that their reaction to LDIR can be mediated by oxidized GC-rich cfDNA fragments. Firstly, the responses of cells to these fragments are identical to those to 10 cGy of radiation. Thus, cfDNA fragments are stress signaling molecules that regulate RAR to LDIR. We demonstrated that the radiation leads to the increase of oxidized cfDNA in the culture medium. We used a genetic construction containing an easy-to-oxidize (G)n repeat to show that the oxidized cfDNA can rapidly penetrate into the cytoplasm and induce a short-term increase in ROS production, a process implemented by the NOX4 oxidase. This, on the one hand, leads to a short-term oxidative modification of nuclear DNA, but, on the other hand, activates antioxidant systems. An increased level of ROS leads to DNA damage and DSBs, but, at the same time, activates DNA repair and minimizes damage. Moreover, 10 cGy of radiation evoke a strong antiapoptotic response.

**Figure 11:**
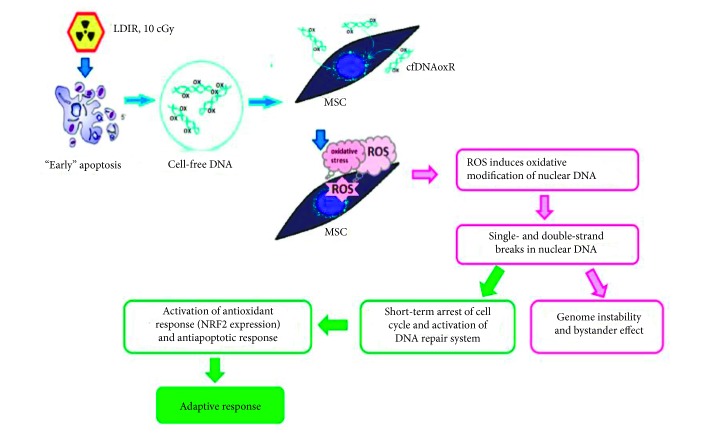
Proposed mechanisms for the development of radioadaptive responses and bystander effect. Irradiation induces primary oxidative stress and oxidation of genomic DNA → apoptosis of some irradiated cells → release of oxidized cfDNAoxR → reactive oxygen species (ROS) production → ROS induces oxidative modifications of nuclear DNA, rapidly repaired DNA breaks, short-term arrest of the cell cycle → activation of DNA reparation systems and antioxidant response → inhibition of apoptosis. Thus, we conclude that cfDNAox that appears after irradiation is a signaling molecule in the stress signaling that mediates radiation-induced bystander effects.

Taken together, these data indicate that the cascade of events in cfDNAox signaling may be the following: irradiation → primary oxidative stress → oxidation of genomic DNA → apoptosis of some irradiated cells → release of oxidized cfDNAoxR → ROS production → ROS induces oxidative modifications of nuclear DNA, rapidly repaired DNA breaks, and short-term arrest of the cell cycle → activation of DNA repair systems and antioxidant response → inhibition of apoptosis → radioadaptive responses (RAR) (Figure 11). We believe that oxidized DNA is one of the components of damage-associated molecular pattern molecules. The supposed mechanism of penetration of our plasmids into cells includes the oxidation of dG (in dG repeats) on the cell surfaces. Oxidized DNA fragments penetrate into cells, as shown previously [59]. When the oxidation is moderate, the unchanged promotor allows the inserted GFP gene to be transcribed. In order to optimize the amount of plasmids in the cytoplasm, we conducted an experiment in conditions with a slightly elevated level of ROS on cell surfaces and in the medium by addition of H_2_O_2_. In these conditions, the fluorescence intensity of the cytoplasm was elevated more than without H_2_O_2_. Thus, the level of GFP fluorescence increases when the cells are under a moderate oxidative stress caused by either radiation or H_2_O_2_. This indicates that cfDNA needs to be oxidized in order to penetrate into the cells.

The secondary oxidative stress that is evoked in bystander cells occurs after interaction of cfDNAox with its receptors on the cell surface or inside, possibly transmembrane proteins of the toll-like receptor family, namely TLR9 [60], whose ligation may lead to the elevation of ROS [60, 61]. Binding of CpG-DNA to TLR9 increases the ROS level in human monocytes, [62] and in neutrophils, it leads to the production of peroxynitrite [61]. In addition, oxidized DNA seems to be a stronger TLR9-stimulating ligand than nonoxidized DNA [18]. As we showed previously, GC-rich cfDNA fragments can activate TLR9 [63]. In this cascade, the formation of the “DNA-TLR9” complex initiates the cellular signaling pathway that leads to an activation of NF-*κ*B [63], which in many different ways augments the biosynthesis of ROS. However, when the TLR9 pathway was blocked in irradiated lymphocytes, there were no substantial changes in the localization of 1q12 loci or in the level of ROS [64], indicating that in addition to oxidized DNA-stimulated TLR9 receptors, cells possess other sensors whose activation leads to the changes in ROS and that RIBE may be regulated through more than one molecular pathway. Evidence pointing at the existence of toll-like receptor-independent stress signal transfer pathways was demonstrated by other authors, including cytoplasmic DNA-dependent STING, AIM2, RIG-1, and DAI sensor pathways [65]. Apart from that, cells may possess a variety of molecules that sense the damage in cfDNA and may respond differentially to oxidized DNA bases. The reception of cfDNAox produced by irradiated cells warrants further investigations.

ROS level increases drastically during the first minutes after the addition of cfDNAox or cfDNAoxR to the medium, but decreases 30 min after the addition. We propose that activation of ROS production is connected to a changed expression of ROS-coding enzyme/enzymes such as the *NOX* family enzymes of NADPH oxidases [24]. One-2 h after treatment, cells have a moderately elevated level of ROS if the DNA is not oxidized. The reaction of MSCs to oxidized DNA is more rapid compared that of differentiated cells [55]. Oxidative stress is the key stage that initiates the cfDNAox signaling pathway which, on the one hand, triggers DNA oxidation and damage in the cells but on the other hand allows the development of the adaptive response (activation of DNA repair, activation of antioxidant transcription factor NRF2, and apoptosis inhibition) (Figure 11). Our evidence suggests that cfDNAoxR that appears after irradiation is responsible for the stress signaling that mediates radiation-induced bystander effects (RIBE) and moreover is an important component of the development of radioadaptive responses (RAR) to low doses of IR. Since they are a pool of reserve cells for critical situations and a tool for tissue regeneration with a relatively high proliferative potential [66], changes in cfDNA properties can be crucial for MSCs because DNA breaks can lead to chromosomal aberrations and insertions of DNA into the breakpoints.

## Figures and Tables

**Table 1:** Sources and characteristics of MSC lines. Cell lines were obtained from the Research Centre for Medical Genetics, Moscow.

Cell	Source	Surface markers			
MSC	Adipose tissue from mammary gland	CD34−	CD45−	HLA-ABC+	HLA-DR−
		CD44+	CD29+	CD49b low	CD54 low
		CD90+	CD106−	CD105 low	CD117−

**Table 2:** Content of 8-oxodG per 10^6^ nucleotides in different types of DNA.

	Content of 8-oxodG per 10^6^ nucleotides
gDNA	<0.01
cfDNAox	400
cfDNAoxR	200

**Table 3:** Dependence of the changes in the levels of antiapoptotic genes *BCL2*, *BCL2A1 (Bfl-1/A1)*, *BCL2L1 (BCL-X)*, *BIRC2 (c-IAP1)*, and *BIRC3 (c-IAP2)* and proapoptotic *BAX* expression in irradiated and exposed to cfDNAox, cfDNAoxR, and gDNA in MSCs on the time after exposure (RT-PCR). mRNA level—average expression of genes in treated MSCs compared to control (for three biological replicates). Reference gene—*TBP*. ^∗^*p* < 0.001, nonparametric *U* test (qRT-PCR).

Gene	Changes in the expression levels, arb.un.				
Treatment	Time	10 cGy	cfDNAox	cfDNAoxR	gDNA
*BCL2*	30 min	2.6 ± 0.3^∗^	2.3 ± 0.2^∗^	2.4 ± 0.2^∗^	1.1 ± 0.2
	2 h	4.1 ± 0.4^∗^	3.1 ± 0.2^∗^	3.3 ± 0.3^∗^	1.0 ± 0.2
	24 h	3.9 ± 0.3^∗^	2.9 ± 0.2^∗^	2.9 ± 0.3^∗^	1.3 ± 0.3
	48 h	2.8 ± 0.3^∗^	3.1 ± 0.3^∗^	2.6 ± 0.2^∗^	1.8 ± 0.3^∗^
*BCL2A1 (Bfl-1/A1)*	30 min	1.6 ± 0.2^∗^	2.4 ± 0.2^∗^	2.5 ± 0.3^∗^	1.0 ± 0.2
	2 h	2.1 ± 0.2^∗^	2.2 ± 0.1^∗^	2.4 ± 0.2^∗^	1.1 ± 0.2
	24 h	2.1 ± 0.3^∗^	2.8 ± 0.3^∗^	2.3 ± 0.2^∗^	1.3 ± 0.2
	48 h	1.8 ± 0.3^∗^	1.6 ± 0.2^∗^	2.2 ± 0.2^∗^	1.7 ± 0.3^∗^
*BCL2L1 (BCL-X)*	30 min	1.4 ± 0.2	1.6 ± 0.1^∗^	1.6 ± 0.1^∗^	1.1 ± 0.2
	2 h	1.9 ± 0.3^∗^	1.5 ± 0.2^∗^	1.7 ± 0.2^∗^	1.2 ± 0.3
	24 h	2.3 ± 0.2^∗^	2.7 ± 0.3^∗^	2.4 ± 0.2^∗^	1.1 ± 0.3
	48 h	2.8 ± 0.3^∗^	2.4 ± 0.3^∗^	1.9 ± 0.2^∗^	1.6 ± 0.2
*BIRC2 (c-IAP1)*	30 min	2.4 ± 0.2^∗^	1.4 ± 0.2	1.8 ± 0.2^∗^	1.0 ± 0.3
	2 h	2.6 ± 0.2^∗^	2.1 ± 0.2^∗^	2.3 ± 0.3^∗^	1.0 ± 0.2
	24 h	2.6 ± 0.3^∗^	2.7 ± 0.3^∗^	2.6 ± 0.2^∗^	1.6 ± 0.3^∗^
	48 h	2.4 ± 0.2^∗^	2.4 ± 0.3^∗^	2.5 ± 0.2^∗^	2.5 ± 0.3^∗^
*BIRC3 (c-IAP2)*	30 min	2.2 ± 0.1^∗^	1.2 ± 0.2	1.2 ± 0.2	1.0 ± 0.2
	2 h	2.5 ± 0.2	1.9 ± 0.2^∗^	1.8 ± 0.2^∗^	1.2 ± 0.3
	24 h	2.5 ± 0.2	2.6 ± 0.3^∗^	2.5 ± 0.3^∗^	1.6 ± 0.3
	48 h	2.0 ± 0.3	2.5 ± 0.4^∗^	2.5 ± 0.4^∗^	2.0 ± 0.4^∗^
*BAX*	30 min	1.6 ± 0.2^∗^	1.5 ± 0.2^∗^	1.4 ± 0.2	1.0 ± 0.2
	2 h	0.8 ± 0.2	0.8 ± 0.2	0.9 ± 0.2	1.1 ± 0.3
	24 h	0.8 ± 0.2	0.9 ± 0.2	1.1 ± 0.2	0.9 ± 0.2
